# The Potential Metalloestrogenic Effect of Aluminum on Breast Cancer Risk for Antiperspirant Users

**DOI:** 10.3390/ijms26010099

**Published:** 2024-12-26

**Authors:** Ewa Sawicka, Natalia Wiatrowska

**Affiliations:** 1 Department of Toxicology, Faculty of Pharmacy, Wroclaw Medical University, 50-556 Wroclaw, Poland; 2 Students’ Scientific Society at the Department of Toxicology, Faculty of Pharmacy, Wroclaw Medical University, 50-556 Wroclaw, Poland; natalia.wiatrowska@student.umw.edu.pl

**Keywords:** endocrine disrupting chemicals, Al, toxicity, antiperspirants, breast cancer

## Abstract

The etiopathogenesis of breast cancer depends on genetic conditions, but recently more attention has been paid to the dependence of BC on certain environmental factors, for example, metalloestrogens, which include aluminum (Al) contained in antiperspirants used daily. The use of Al derivatives in antiperspirants in concentrations specified by the FDA, as well as European regulations (SCCS, 2020), do not classify Al as a hazardous and carcinogenic substance for humans. However, Al used to treat excessive sweating raises concerns, as many in vitro studies indicate that it can cause gene instability, change gene expression or increase oxidative stress, and also affect the body’s hormonal balance as a metalloestrogen. The environmental reality is that the breast is constantly exposed to many different chemicals, such as Al. This article reviews the literature to determine whether Al-based products can harm the body, as there are many facts and myths on the subject. The aim of the study is to present the current state of knowledge on the use of aluminum antiperspirants and the risk of breast cancer (BC). The article is based on data from the scientific literature, published in the PubMed and Google Scholar databases, as well as Science Direct, Scopus, Ovid MEDLINE, Ovid EMbase. It includes articles published in the years 2003–2023 mainly in English. Literature databases regarding human and animal studies were searched. To sum up, evaluating the effect of Al as a risk factor for breast cancer requires many studies using different research models focused on long-term exposure to Al-containing antiperspirants. Consumers are advised to limit their exposure to Al by making a conscious choice to minimize exposure to this compound.

## 1. Introduction

Breast cancer (BC) is one of the most common cancers in women. The etiopathogenesis of breast cancer depends on genetic and hormonal conditions, but more and more attention is focused on the dependence of BC on certain environmental factors [1,2,3]. These factors are referred to as xenoestrogens, or so-called endocrine-disrupting chemicals (EDCs). Binding of EDCs to estrogen receptors (ERs) results in activation of estrogen signaling pathways. There are intracellular ERs, including estrogen receptor alpha (ERα) and estrogen receptor beta (ERβ), as well as membrane-bound ERs, such as membrane-bound ERs (mERs). In addition to binding to ERs, exogenous estrogens bind to membrane-bound growth factor receptors, such as human epidermal growth factor receptor (EGFR/HER) and insulin-like growth factor receptor 1 (IGF1R), as well as nuclear receptors [4,5]. The number of EDCs is very wide, and their action occurs through disruption of the body’s homeostasis in a hormone-dependent manner, which may contribute to the development of breast cancer [5,6,7]. The classification of xenoestrogens depends on the chemical structure of these compounds. This group also includes metalloestrogens, e.g., cadmium (Cd), Al, hexavalent chromium [Cr(VI)] and trivalent chromium [Cr(III)]. Most of the metalloestrogens are released into the environment from industrial sources (mining, metallurgy, electroplating). Humans are exposed to some of these metals in significant amounts in everyday life, e.g., cadmium occurs in tobacco smoke, chromium (III) in dietary supplements used to lower blood glucose levels, and Al mainly in deodorants and antiperspirants. Due to their hydrophobic nature, xenoestrogens accumulate mainly in adipose tissue and then are included in cellular metabolism. Moreover, they can also be passed on to offspring during pregnancy or lactation [8,9,10].

Epidemiological data indicate, however, that the role of certain environmental factors in the incidence of breast cancer remains undetermined. These factors include Al, the presence of which in antiperspirants is particularly controversial because it is applied directly to the skin near the breasts [11]. It is suspected that our daily exposure to Al may affect the development of BC, but the US Food and Drug Administration (FDA) and other health organizations, including the World Health Organization (WHO), state that there is currently insufficient evidence to clearly link Al with breast cancer [12]. The use of Al derivatives in antiperspirants at concentrations specified by the FDA, as well as European regulations do not classify Al as a substance hazardous to human health [13]. According to the American Society for Testing and Materials (ASTM), Al compounds used in cosmetics and personal care products are Al oxide, Al hydroxide, Al hydrochloride, Al citrate, Al myristate and Al distearate [14]. Although the FDA has approved the use of the above Al compounds in cosmetics and personal care products, further studies are recommended to better understand the potential risks associated with their use for human health. Al has not been classified as a carcinogen, but the International Agency for Research on Cancer (IARC) has determined that its production process is carcinogenic to humans [15]. This review presents essential information on Al, as well as the current knowledge on the association between everyday Al exposure and the risk of breast cancer, combined with a critical perspective on this topic.

The aim of this secondary research was to review the available literature data on the molecular mechanisms of Al action on the mammary gland, and especially the effects of long-term exposure to Al, which is present in antiperspirants, on the possible development of breast cancer. Moreover, this key issue was raised because there is still controversy in the scientific community regarding Al as a factor causing breast cancer. The presented article was created based on a literature review, in which the PubMed and Google Scholar databases were used, as well as Science Direct, Scopus, Ovid MEDLINE and Ovid Embase. It includes works published in the years 2003–2023 in English, and the search terms used were: xenoestrogens, Al, toxicity, antiperspirants, breast cancer both in human and animals.

## 2. Aluminum Absorption, Occurrence in the Environment and Sources of Exposure

The skin surface, respiratory system and gastrointestinal tract are the main routes of Al absorption. Al compounds (from antiperspirants) applied topically to the skin are transported through the stratum corneum by passive diffusion. Apocrine and eccrine sweat glands located in the stratum corneum, as well as hair follicles, increase the penetration of Al compounds into the subsequent layers of the skin, i.e., the epidermis, dermis and subcutaneous tissue. In this way, aluminum compounds from topically applied cosmetics enter the bloodstream. It is possible that Al is partially stored in the skin structures and that its ions enter the lymphatic system. The nature of Al compounds present in topically applied cosmetics, as well as the considerable frequency and regularity of their use, mean that the skin is a permanent source of biologically available Al, both locally and systemically [16]. Al enters the lungs in the form of aluminosilicate particles and compounds that are poorly soluble in water. Particles are absorbed by phagocytosis and in this form some of them are transported through the respiratory tract epithelium to the larynx and lungs, which also constitute a systemic reservoir of Al [17]. In contrast, absorption from the intestines is a two-stage process: absorption of Al from the intestinal lumen to the mucosa and a slower second stage, from the mucosa to the blood, where the absorbed Al binds to plasma proteins (70–90%), mainly transferrin. Al is moved from the blood to the tissues quite quickly. Al is excreted from the body mainly by the kidneys (approx. 30 µg/24 h) [18]

Al exposure comes from diet, antacids and vaccine adjuvants, but the frequent use of Al-based compounds under the arms as antiperspirants results in significant additional exposure directly to the local surface of the human breast [16]. Evidence suggests that the upper outer quadrant of the breast also has a disproportionately high incidence of breast cysts and breast cancer. Cystic breast disease is the most common benign breast disease, and evidence has been produced that Al may be a causative factor in the development of breast cysts. It may also cause genomic instability and inappropriate proliferation in human breast epithelial cells, resulting in increased migration and invasiveness of breast cancer cells. Al, as a metalloestrogen, affects ERs and may thus become an additional risk factor for breast cancer [16].

About 95% of the daily intake of Al is supplied with food, and about 1–2% with drinking water. The average daily intake of Al from various sources is 4000–9000 µg [19]. High Al content has been observed in beverages stored in cans (5–7 times higher compared to the same beverage stored in bottles). Potential sources of Al ions are gastric acid-neutralizing drugs containing Al (35–200 mg/dose), and their use can lead to an increase in Al intake of up to 2000 mg daily [18,19]. Other sources of Al include toothpastes, UV filters, disinfectants, fumigants, pesticides, antiperspirants and cosmetic products, including for make-up (e.g., Al content in eye shadow is 20,000–50,000 µg/g, in lipstick 14.2–27,032 µg/g, in face cream 170–650 µg/g, in hand cream 5400–8500 µg/g) [20,21,22]. Antiperspirants containing Al compounds may increase their daily intake even to 50,000–75,000 µg [23].

**Figure 1:**
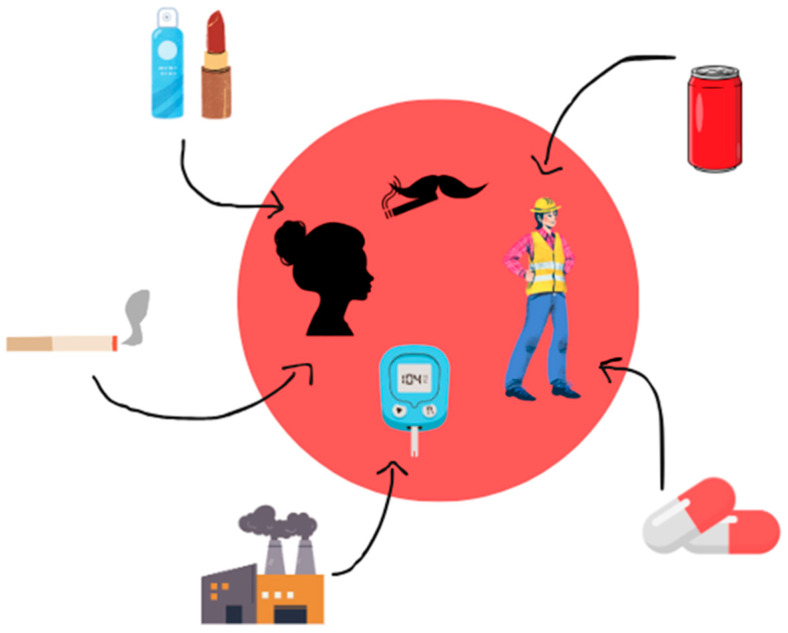
Sources of Al that affect the human body. Own work based on [18,19].

Exposure to Al can come from various sources, including cosmetics, food, cigarette smoke and industry. At-risk groups, such as women using cosmetics, diabetes patients, workers in the Al industry and smokers, should be aware of the potential hazards associated with this metal (Figure 1).

Increased concentration of Al in blood may occur in workers of the industry related to Al processing, as well as in patients taking parenteral drugs and in patients undergoing dialysis procedures. It has been shown that dialysis membranes do not eliminate Al as effectively as kidneys. This was found to result in the accumulation of Al ions in bone tissue (their concentration was 100 mg/kg of dry mass, while in non-dialysis patients it was 27.4 mg/kg of dry mass), which due to their effect on osteoclasts, manifested itself in the occurrence of osteomalacia and osteodystrophy. The FDA specifies the maximum dose of Al, which, when delivered during parenteral nutrition, does not cause excessive accumulation of this ion. It should not exceed 4–5 µg/kg/day. Chemical substances containing Al in their molecules are widely used in various products and processes related to human activity. These compounds include Al chloride, Al hydroxide (Al oxide trihydrate), Al nitrate, Al phosphate, Al sulfate, Al potassium sulfate, Al ammonium sulfate and Al silicate [23,24]. The Al content in the diet of adults in different countries is presented in Table 1 [25,26].

## 3. Aluminum Toxicity

There are countless possibilities of binding Al in every biological environment because it shows a tendency to participate in biochemical processes, especially through strong binding of functional groups containing oxygen. Al is a strong oxidant, and by participating in free radical reactions, it may play a role in the induction of oxidation-reduction disorders, leading to oxidative stress, the role of which in the pathogenesis of cancer is well documented. Al compounds in the human body react with metal ions necessary for the functioning of the body (e.g., magnesium, calcium and iron), thus changing their bioavailability [25,26]. Al salts are used as phosphate binders and are added to dialysates. Exposure to Al compounds in dialysis patients resulted in an increased concentrations of this metal ion in plasma and brain, which manifested itself as disorientation, memory disorders and, in advanced stages, dementia. Al binds to the structures of the neuronal cell membrane and modifies signaling pathways in the hippocampus. The above-mentioned actions influence the neurotoxic effects of this metal [27]. Neurological disorders, such as Alzheimer’s disease (AD), Parkinson’s disease and dialysis encephalopathy, have been associated with Al toxicity. Exposure to high levels of Al leads to neurofibrillary degeneration, and it has been observed that neuronal Al concentrations increase in AD. However, the role of Al in AD remains controversial and there is little evidence to directly link Al to AD. Administration of Al maltolate to New Zealand white rabbits resulted in pathology in the form of neuropathological, biochemical and behavioral changes characteristic of AD. Neurodegenerative effects included the formation of intraneuronal tau-positive neurofilament aggregates, as well as the generation of oxidative stress and apoptosis [28].

The main mechanism of toxicity of Al, as already mentioned, consists in the disruption of metal homeostasis. The physical and chemical properties of Al and its affinity for oxygen atoms, hydroxyl or phosphate groups means that Al can effectively imitate the above metals and their corresponding biological functions, disturbing many processes in the body [18]. Studies have shown that exposure to Al contributes to abnormalities in bone structure, mammary gland disorders and pulmonary granulomatosis. Al is also associated with the initiation and maintenance of chronic, recurrent intestinal inflammation in people genetically predisposed to Crohn’s disease [29,30].

It is important to control the levels of Al by using biomonitoring (BM). It can help prevent the accumulation of Al in target organs, identify exposed individuals and roughly quantitate transient exposure. Urinary Al (U-Al) should be collected two days after exposure and its value was found to correspond roughly to 2.3 µmol/g creatinine [31]. The results showed that the level of Al in urine was higher in Al workers compared to the controls [32].

## 4. Al as a Metalloestrogen and Its Potential Role in the Pathogenesis of Breast Cancer

**Figure 2:**
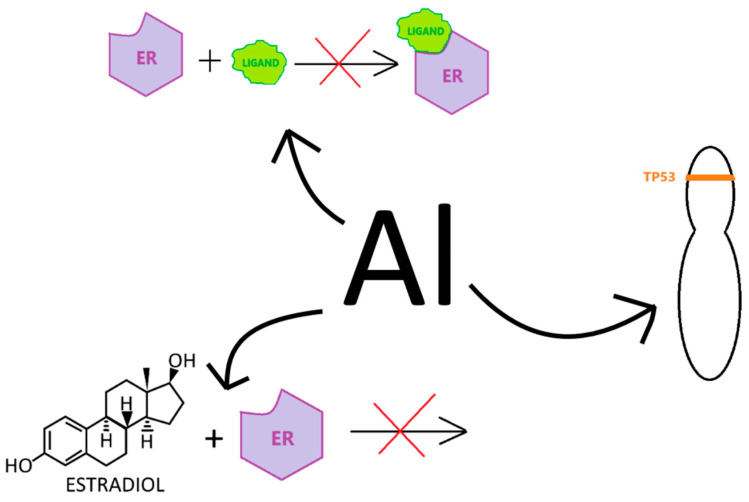
Impact of Al on cellular processes. Al disrupts cellular function by preventing estradiol from binding to ER and inhibiting the formation of receptor–ligand complexes. It has the ability to regulate the expression of genes crucial for normal cell growth; TP53 is an example of such a gene—it encodes the tumor protein p53 which regulates the cell cycle and maintains genomic stability. Additionally, Al interferes with the binding of estradiol to ER. All of these disruptions can lead to developing breast cancer. Own work based on [9,35,36].

One of the subgroups of xenoestrogens, metalloestrogens, can disrupt the functioning of the hormonal system by affecting ERs. In addition to the above-mentioned ones, i.e., cadmium, chromium and Al, there are also lead, copper, cobalt, nickel, mercury, tin ions, vanadium and arsenate anions [5,9,33]. The above metalloestrogens are subject to bioaccumulation, and as a consequence, serious damage to the body can occur, as well as the development of cancer [33,34]. Al as a metalloestrogen interacts with ERs, disrupting their functioning by preventing estrogen from binding to ERs and the formation of receptor–ligand complexes. Al may interfere with the function of estrogens, hormones that play a key role in the development of breast cancer (Figure 2) [9,35,36].

In breast cancer cells, there are many genes regulated by estrogens, both through activation and inhibition. The function of these genes can be changed by metalloestrogens. For example, Al chloride can interfere with the function of ERs and estrogen binding to ERs and disrupt the ability of the ligand–receptor complex to bind to estrogen response sites. Al can also change the cellular profile by regulating the expression of a gene necessary for normal cell growth. Al compounds can interfere with the binding of estradiol to ERs in normal cells, but the exact mechanism of this process is still not fully understood. Tests have also been carried out to examine the effect of Al on the estrogen-dependent breast adenoma cell line MCF-7 [35]. It was shown that Al can interfere with the action of steroid hormones in a way typical for xenoestrogens. Currently, the aim of many scientific studies is to understand the molecular mechanisms of these processes and the effects of long-term action of Al on the possible development of breast cancer, changes in the DNA structure and in cell signaling pathways. This is extremely important for the prevention of breast cancer in women exposed to long-term use of antiperspirants, which contain Al, constituting even one quarter of the volume of the cosmetic. Applying deodorant in the area of the mammary gland, often on skin freshly irritated by shaving, facilitates the penetration of Al into the bloodstream, resulting in the stimulation of mammary gland cancer cells. Scientific studies have shown that Al affects ERs, inducing gene expression, but more research is still needed on the subcellular action of Al on breast cells [36,37].

Darbre et al. [35], in their studies, showed that Al in the form of aluminum chlorohydrate (ACH) interfered with the functioning of ERs. Moreover, they indicated that long-term exposure of human breast epithelial cells MCF-10A to Al chloride or Al chlorohydrate may cause a decrease in mRNA levels. In this study, it was also found that the decrease in mRNA levels for several other key DNA repair proteins, including BRCA2 (Breast Cancer Gene 2), CHK1 (Checkpoint kinase 1), CHK2 (Checkpoint kinase 2), Rad51, and ATR, was caused by long-term exposure to Al. Other studies have confirmed the effect of ACH on reducing the level of ERβ protein, which may be related to the increase in the concentration of ERα. Decreased expression of ERβ contributes to the weakening of their anti-apoptotic and antioxidant activity, which hypothetically may result in the acceleration of the development of breast cancer under the influence of Al [16,38].

**Figure 3:**
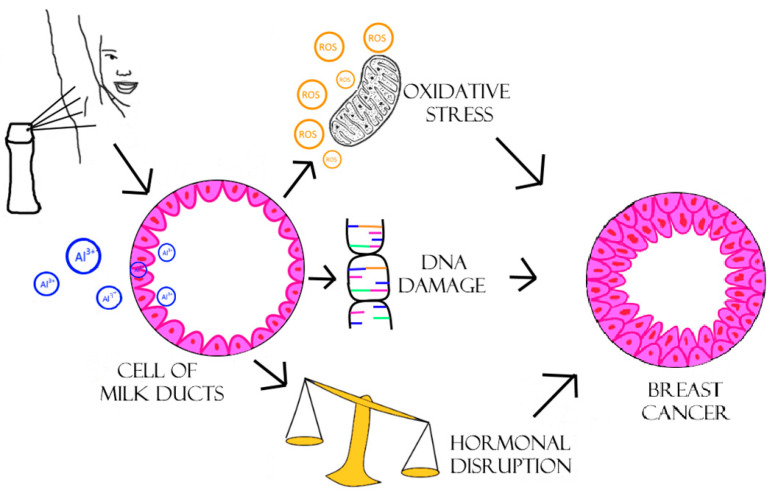
The effect of Al-based deodorants on milk duct cells. Al ions penetrate the cells, inducing oxidative stress, DNA damage, hormonal disruption and excessive proliferation. Own work based on [43,45].

One of the most frequently cited studies on the toxic effects of Al contained in antiperspirants on the human body was conducted by Gulliard et al. [39], presenting the case of a patient complaining of severe bone pain and chronic fatigue. All routine biochemical tests performed on the patient gave results within the norm; therefore, the diagnostics were extended to include tests of heavy metal concentration levels. They showed high hyperaluminemia, amounting to 3.88 mol/L (normal range: 0.1–0.3 mol/L). The interview with the patient showed that for the past 4 years, she had been using a local antiperspirant every day, on regularly shaved armpit skin. The search for other possible sources of Al excluded the consumption of medications containing significant amounts of Al (e.g., antacids) or daily exposure related to professional work (librarian). It was shown that the source of Al was the daily, regular use of antiperspirant. Discontinuation of Al use for eight months resulted in a significant reduction in plasma Al concentration to values within the normal range and, consequently, all symptoms disappeared [39]. In order to find the possible influence of Al on the development of breast cancer, a study was carried out in which volunteers were studied and divided into two groups: the first group consisted of healthy women referred to as the control group, while the second group consisted of women diagnosed with breast cancer within the last five years. Breast tissue biopsies were taken from both volunteers and patients, and the concentration of Al in the biopsy material was determined. Analysis of the samples showed that Al was present in the tissues of both healthy patients and patients with breast cancer, and the more often the women used antiperspirants, the higher the concentration of Al in them. In patients with breast cancer, the average concentration of Al in breast tissue was 5.8 nmol/g, and in healthy patients it was 3.8 nmol/g of tissue [40]. In experiments conducted by Gorgogietas et al. [41], the effect of 4 h exposure to ACH at a concentration of 10-4 M on the estrogen-dependent breast cancer cell line MCF-7 was assessed by examining the level of ERα protein. During the experiments, a 2–3-fold increase in ERα protein concentration was observed compared to the control sample not treated with the tested Al compound. This indicates the estrogenic activity of Al and its participation in the molecular mechanism causing the ACH-induced increase in receptor activity [41]. The effect of 17β-estradiol (E2) and ACH on the synthesis of ERα mRNA was also assessed to determine whether the effect of ACH on the level of estrogen receptor protein is dependent on the activation of ERs gene expression. After 6 h of exposure of MCF-7 cells to 10-9 M E2 and ACH at a concentration of 10-4 M, a significant induction of ERα mRNA synthesis was observed. This effect may also suggest that Al has a significant effect on the stability of the estrogen receptor protein. In the above study, it was also shown that ACH can affect the expression of p53, p21 or cyclin D1 genes, which are key regulators of breast cancer progression and/or cell senescence. The expression of these genes was examined in the MCF-7 breast cancer cell line (estrogen-dependent) and the triple-negative breast cancer line-MDA-MB-231 after exposure to ACH and E2. ACH-induced cyclin D1 gene expression may be to some extent dependent on ERα, because this effect was abolished in ERα-negative MDA-MB-231 cells. The authors showed that long-term exposure of MCF-7 cells to Al salts, such as Al chloride (AlCl3) and ACH at a concentration of 10-4 M, caused an approximately two-fold increase in the activation of estrogen response processes [41]. Namely, results from the studies presented above showed a higher Al content was observed in breast tumors than in healthy tissues (1.5–3.5 times more) [40,41]. In addition, a higher Al content was also shown in tumors of the outer part of the region of this organ. The most common finding is that Al applied to the skin after using an antiperspirant accumulates in the mammary gland tissue, mainly in the external area, and thus can induce tumors, where higher Al concentrations have been observed than in healthy tissue. In addition to Al, significantly increased concentrations of other metal ions have been observed in human breast cancer tissue, e.g., As, Ca, Cd, Cl, Co, Cr, Cs, Cu, Fe, K, Mn, Na, and Pb [42,43]. Some of these metal ions, e.g., As, Cd, Co, Cr, Cu, Fe, Pb and Zn, play a role in mammary gland carcinogenesis in in vitro models, but also in studies on experimental animals via estrogenic mechanisms. Oxidative damage in the cell can also occur and, as a result, destruction of signal transduction pathways of cancer cell proliferation and migration. It is assumed that the synergistic effect of metals, often with carcinogenic potential, may have a potential impact on carcinogenesis (Figure 3) [44,45].

Based on the above data, it can be concluded that frequent use of Al-based compounds under the arms as antiperspirants causes additional exposure directly to the local surface of the human breast. The concentration of this metal ion measured in human breast tissues and fluids showed higher level than in blood, and experimental evidence suggests that at physiological concentrations Al may adversely affect the biology of human breast epithelial cells. The observed cystic breast disease is one of the most common benign breast diseases. Studies indicate that Al may be a factor facilitating the development of breast cysts. Al, in particular, may cause genome instability and inappropriate proliferation in human breast epithelial cells, increasing their invasion. Based on the literature reports cited above, it has been shown that Al as a metalloestrogen may be a risk factor for breast cancer. The microenvironment has been considered another factor determining the development of breast cancer and it has been shown that Al causes adverse changes in the breast microenvironment contributing to the development of breast cysts and breast cancer [46]. The human breast microenvironment consists of the extracellular matrix (ECM), epithelial cells, fibroblasts and adipocytes. These cells are surrounded by various ECM molecules that play a key role in physiological remodeling and may stimulate breast carcinogenesis. The results of the studies support the possible involvement of Al ions in the perturbation of the oxidative and inflammatory state of the breast cancer microenvironment, suggesting that Al accumulation in the breast microenvironment is a possible risk factor for the oxidative/inflammatory phenotype of breast cells [47].

In the case of breast cancer, the National Statistics Offices for England, Wales and Scotland have been collecting information on the location of breast cancer since 1979 and these data indicate an increasing incidence of breast cancer in the upper outer quadrant of the breast compared with other breast areas over recent decades. This increasing incidence in one particular breast area cannot be explained solely by the greater amount of epithelial tissue in this region, but the hypothesis of an association between increased underarm antiperspirant use and breast cancer still merits serious investigation. The main salts used are complexes such as Al chloride, Al chlorohydrate and Al-zirconium chlorohydrate complexes with glycine. In the European Union (EU), Al zirconium hydroxide is permitted in antiperspirants up to 20% in anhydrous form. However, the Cosmetics Directive additionally states “do not apply to irritated or chapped skin”, which is contrary to current practices of shaving the skin prior to applying antiperspirant. The general public may not be aware that shaving is a procedure that can cause skin abrasion, loss of the stratum corneum and irritation due to depilation, but this common practice contradicts the detailed recommendations in the EU Cosmetics Directive. Furthermore, it can be anticipated that such skin damage due to shaving procedures would allow for greater absorption of Al into the underlying tissues. The extent to which such continuous exposure combined with prior shaving could lead to absorption of Al at low levels in the longer term into the underlying breast tissue is of current interest to researchers [33]. Example studies on the effect of Al in cell line models (in vitro), in animal studies (in vivo) and epidemiological data from breast cancer patients with concomitant exposure to Al ions are presented below (Table 2).

## 5. Controversies Between Al Exposure and Breast Cancer

A thorough systematic analysis of the correlation between daily exposure to Al and the risk of breast cancer by Moussaron et al. [57] showed that some studies on the association between the use of deodorants and antiperspirants and the occurrence of breast cancer did not produce consistent results. Among 13 studies on Al content in breast tissues and the risk of BC, the results were not clear in assessing the higher Al content in tumor tissues compared to healthy tissues. They were conducted in small cohorts and without long-term follow-up. On the other hand, studies on cell lines have shown the carcinogenic potential of Al. Moreover, BC was considered a separate group in the studies, while BC is a heterogeneous disease in which there are many subtypes of cancer determining the aggressiveness of the tumor. The authors of the above data analysis conclude that in the light of the precautionary principle and the obtained data, it is better to avoid antiperspirants containing Al [57].

Klotz et al. [26] consider whether there is a link between Al exposure and breast cancer. The researchers report that analysis of 746 consecutive breast tissue samples showed comparable rates of benign or malignant lesions diagnosed and were also comparable between the individual breast quadrants. Thus, according to the authors, Al does not appear to be a tumor trigger, but instead indicates that Al is stored to a greater extent in tumor tissue than in healthy tissue [26]. A more recent study showed that long-term exposure to Al chloride caused transformation of breast epithelial cells in vitro, e.g., through increased DNA synthesis and double-strand DNA breaks, which formed tumor and metastatic cells in animal studies, which may be considered evidence of cellular transformation. A retrospective study found an earlier age at disease onset in breast cancer patients who used Al-containing antiperspirants in combination with underarm shaving, but case-control studies did not identify an association between antiperspirant use and breast cancer risk. The authors of the review state that there are currently no consistent data from epidemiological studies on the association between Al exposure and breast cancer risk [26].

The controversy surrounding Al ions as a factor causing breast cancer results from different interpretations of results and a lack of clear evidence, especially from studies of women diagnosed with breast cancer. In particular, population studies comparing the use of antiperspirants with the risk of breast cancer are ambiguous. Unfortunately, they do not take into account other factors, such as genetic predisposition or lifestyle. The presence of Al in mammary gland tissues, determined by many researchers, does not indicate that it is a causative factor of breast cancer. This may result from the accumulation of various risk factors, including hypothetically, Al. Some authors clearly suggest that the Al content of breast tumors was not significantly different from that of the surrounding normal breast tissue. These authors did not detect significant differences in Al concentrations in relation to the location of the breast tumor, or to important tumor characteristics, such as stage and size. It is, therefore, important and of understandable scientific interest whether Al concentration is associated with key genomic abnormalities associated with breast carcinogenesis, and this has been confirmed in many in vitro models [58].

## 6. Summary

The above studies indicate that Al is widely distributed in the environment, is easily absorbed into the human body and can have toxic effects. Increased Al content, among others in cosmetics, may have a hypothetical effect on the development of breast cancer, and reducing the concentration of Al in cosmetics is an issue indicated as important for introduction into current national/European regulations. Some studies have shown increased concentrations of Al in the tumor or in the peritumoral zone in patients with breast cancer. The environmental reality is that the breast is constantly exposed to many different chemicals, including metals, and usually for many years, as there are often decades between exposure to a carcinogen and the development of cancer. It is still prudent to limit exposure to Al in antiperspirants and deodorants, and above all, to avoid using these products on damaged or irritated skin, especially after activities such as depilation. Better and broader collection of epidemiological data, toxicological studies or the discovery of specific biomarkers, i.e., measurable changes in the body’s cells caused by absorbed Al, are of great importance in the prevention of breast cancer. However, this still requires a lot of experience in the use of different research models, because so far there has been no confirmation of adverse, carcinogenic effects of Al ions. Consumers are advised to limit their exposure to Al by making informed choices about consumer products. Although research is ongoing, there is no clear evidence that Al causes breast cancer. However, for those who want to minimize the risk, avoiding products containing Al can be considered a precautionary measure. There are a number of confounding factors that can affect breast cancer. Some of these include hormone therapy, exposure to chemicals such as pesticides, phthalates, and other chemicals that may be associated with increased risk. In addition, mutations in genes such as BRCA1 and BRCA2 can significantly increase the risk of developing breast cancer. Understanding all these issues is key to fighting breast cancer [59].

Finally, the research into the effects of Al on breast cancer is a topic that requires further study because of the growing interest in the potential risks and health effects of Al exposure. Following are some suggestions for future research directions, recommendations, and potential limitations: expand research on the molecular mechanisms by which Al may affect breast cancer development and progression, including interactions with estrogen receptors and damage by reactive oxygen species; conduct large-scale epidemiological studies that could determine the association between aluminum exposure (e.g., deodorants, foods) and breast cancer risk. Also, use a variety of research methodologies, such as genomics, proteomics and metabolomics, to better understand the effects of aluminum on the body. Unfortunately, potential limitations include the complexity of biological interactions and factors influencing breast cancer development, which may obscure research results. Increased scrutiny of aluminum use in consumer products is certainly a recommendation. Studies on the effects of aluminum on breast cancer can provide important information but require careful design and interpretation of results. Interdisciplinary collaboration will be essential to obtain reliable and comprehensive results [60,61].

## Figures and Tables

**Table 1:** Adult dietary intakes of Al.

Country	Level of Al	Reference
Australia	1.9–2.4 mg/day	Klotz et al., 2017 [26]
Finland	6.7 mg/day	
Germany	8–11 mg/day	
Japan	4.5 mg/day	
The Netherlands	3.1 mg/day	
Sweden	13 mg/day	
Switzerland	4.4 mg/day	
USA	7.1–8.2 mg/day	
Poland	0.6–15 mg/day	Exley et al., 2013 [25]

**Table 2:** List of experiments and epidemiological studies on the effect of Al on breast cancer.

Experiment Cell Line/ Experiment Population/Experiment Animal	Al Formulation/ Dosage	Analysis Method	Result	Reference
Experiment cell line				
MCF-7 human breast cancer cell line	Al chlorohydrate 1 μM 10 μM 100 μM	6 h exposure	The increase in estrogen receptor alpha (ERα) protein levels, in ERα-positive MCF-7 cells. The increase in the mitochondrial Bcl-2 protein. Al actions on ERs protein level and subcellular localization possibly affect receptors-mediated actions and thus, Al interference with estrogen signaling	Gorgogietas et al., 2018 [41]
MDA-MB-231 human breast cancer cell line	Al chloride 250 μM	72 h and 21-day exposure	The evaluation on migration and invasion of MDA-MB-231 human breast cancer cells. Al chloride did not change significantly the migration and invasion ability of MDA-MB-231 cells following, both 72 h and 21-day exposure	Roszak et al., 2020 [48]
MCF-7 human breast cancer cell line	Al chloride 100 µM or various concentrations of Al phthalocyanine tetrasulfonate	1-week exposure or 32- to 37-week exposure or 24–72 h exposure	Only long-term exposure to Al can increase MCF-7 human breast cancer cells’ migratory and invasive activity	Darbre et al., 2013 [49], Lee et al., 2015 [50]
NMuMG nontransformed mouse mammary gland epithelial cell line	Al chloride hexahydrate 100 mM, 30 mM or 10 mM	Incubation with three concentrations of Al chloride hexahydrate	Tumor formed—Continuous exposure of mammary epithelial cells to Al enables them to evade the immunological barrier	Mandriota et al., 2016 [51]
Experiment animal				
Female Wistar rats—48 females, Female Sprague-Dawley rats	Al oxide, Al chloride: 64 mg/kg body weight, 128 mg/kg body weight, 256 mg/kg body weight and 200 mg/kg body weight	Intratracheal instillations; 90 days exposure; 30 days exposure	Statistically significant increases in benign and/or malignant lung tumors were noted with the types of Al compounds used in the study. The experiment showed that Al chloride induced problems in rat fertility, increased oxidative stress and caused hormonal disruptions. Al can enhance the effects of N-nitroso-N-methylurea (NMU) in inducing breast cancer.	Pott et al., 2005 [52], Fu et al., 2014 [53], García-Alegría et al., 2020 [54]
Adult zebrafish	Al chloride 10 mg/L	15 days exposure	Behavioral changes, increased oxidative stress markers, protein levels declined	Capriello et al., 2021 [55]
Experiment population				
Study group: 209 women diagnosed with breast cancer within 5 years. Control group: 209 women not diagnosed with cancer within 5 years average age 20–85 years		Personal interview based on an individual questionnaire	The relationship between antiperspirant use and the concentration of Al in breast tissue, as well as the risk of breast cancer, was studied. The level of Al in the interquartile part of the breast was higher than in the control group	Linhart et al., 2017 [40]
Study group: 54 women with breast cancer Control group: 50 women with other diseases but without breast cancer		Personal interview based on an individual questionnaire	There was no relationship between the use of antiperspirant and breast cancer; the incidence in the group burdened with familial breast cancer was higher than in the control group	Fakri et al., 2006 [56]
Women diagnosed with breast cancer–study group 803 (age 20–74). Control group: 783 women without breast cancer		The interview asked about the use of antiperspirants and how often the armpits were shaved before applying the cosmetic.	Showed that there was no link between antiperspirant use and breast cancer risk. Shaving before using them did not increase the risk of breast cancer	Sanajou et al., 2021 [22]

## Data Availability

Not applicable.
